# A Giant Adrenal Mass in a Super Obese Patient

**DOI:** 10.7759/cureus.1572

**Published:** 2017-08-16

**Authors:** Gabriel O Ologun, Zinal M Patel, Adeolu Adeboye, Mounika Guduru, Douglas Trostle, Thomas Vandermeer, David Bertsch

**Affiliations:** 1 General Surgery, Guthrie Clinic/Robert Packer Hospital; 2 Medicine, Winthrop University Hospital; 3 Surgical Oncology, Guthrie Clinic/Robert Packer Hospital

**Keywords:** adrenal mass, pheochromocytoma, retroperitoneal mass

## Abstract

Giant pheochromocytomas (Pheo) are rare entities requiring clinical suspicion coupled with strategic diagnostic evaluation to confirm the diagnosis. The majority of cases are discovered incidentally. The diagnosis consists of biochemical evaluation and imaging study to localize the mass. Pathological examination confirms the diagnosis. The female patient in this case report presented with chest pain, palpitation of three weeks duration and was found on evaluation to have an abdominal mass concerning for pheochromocytoma. She was treated with surgical resection. The pheo measured 20.5 x 18 x 10 cm and weighed 2,582 grams. Pathological examination confirmed the diagnosis of pheochromocytoma.

## Introduction

Pheo is a rare catecholamine-secreting tumor that arises from chromaffin cells in the adrenal medulla. The annual incidence of pheo is about two million and prevalence in the population is 1:6500 [1]. Patients classically present with severe hypertension, episodic headaches, sweating and palpitations [1-3]. Pheo is diagnosed biochemically and localized using different imaging modalities. The definitive management is surgical resection. Here, we report a case of a giant 20.5 cm pheochromocytoma in a super obese female. Informed consent was obtained for the case report, images and for publication.

## Case presentation

A 55-year-old woman with a history of abdominal pain of three weeks duration, chest pain, palpitation, dyspnea on exertion and dizziness was referred to our surgical outpatient clinic upon detection of large abdominal and pelvic masses on computed tomography (CT) concerning for adrenal and uterine in origin respectively. Physical examination revealed systolic blood pressure in 170 and sinus tachycardia with pulse rate of 105, body mass index (BMI) 54 kg/m^2^. Initial laboratory investigations revealed white blood cell (WBC) count of 12.7 K/uL, serum glucose 248 mg/dL. The patient has a history of diabetes mellitus type II and hypertension.

**Figure 1:**
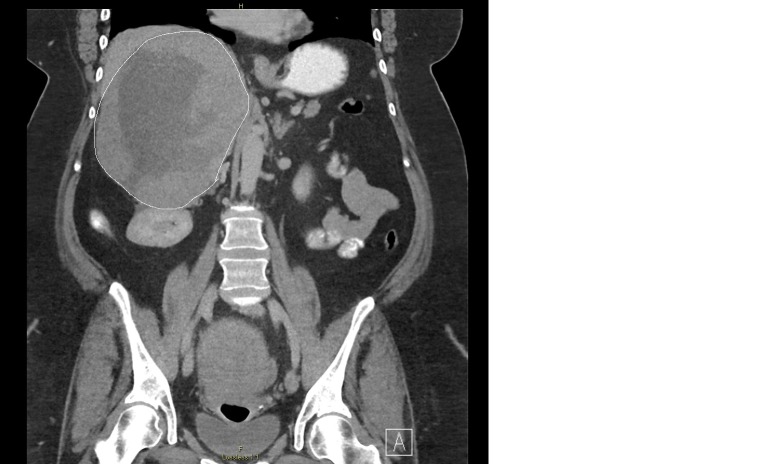
Computed tomography (CT) revealed a 19 x 14.5 x 15.8 cm mass (outlined) involving the right adrenal gland with peripheral heterogenous soft tissue attenuation and low central attenuation with scatter internal coarse calcification, probably with central necrosis or hemorrhage and dystrophic calcification with considerable mass effect on the liver and kidney.

CT revealed a 19 x 14.5 x 15.8 cm mass involving the right adrenal gland (Figures 1).

**Figure 2:**
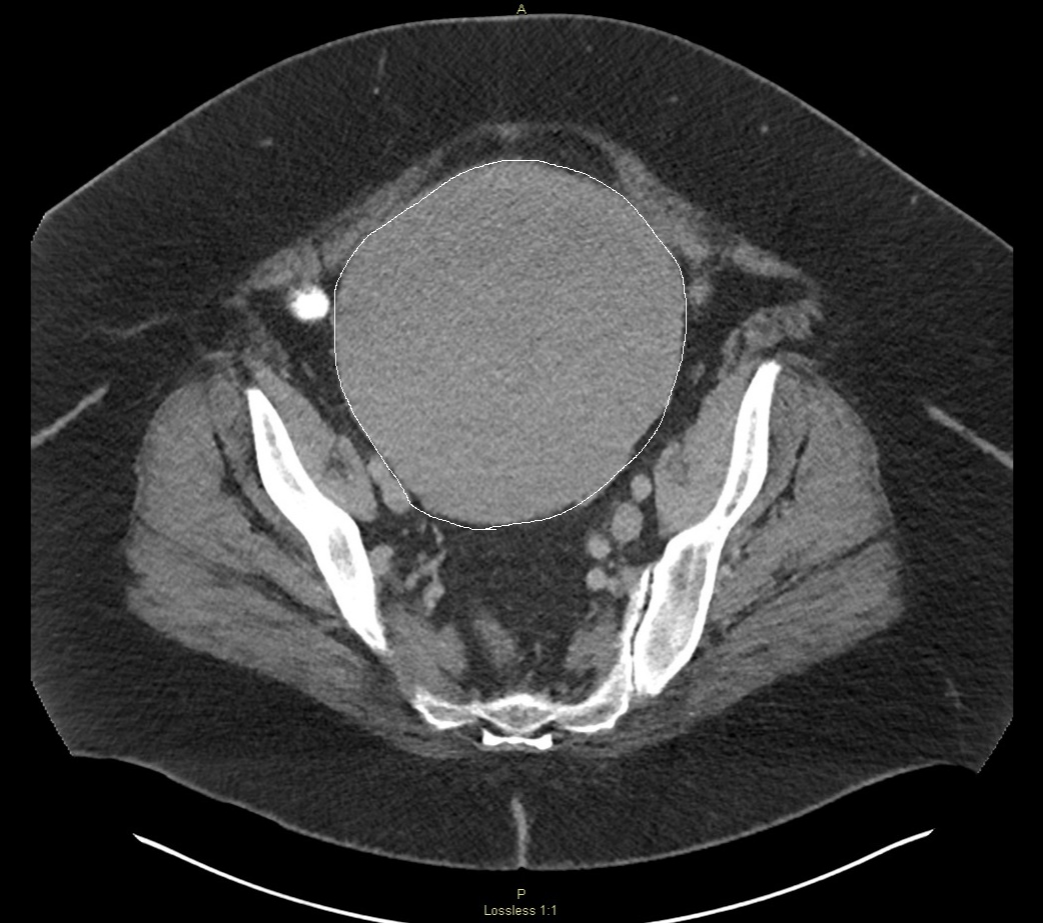
Computed tomography (CT) axial view of the pelvis demonstrating an abnormal mass in the pelvis (outlined) which appears to arise from the uterus with bulbous soft tissue expansion of the organ diffusely measuring approximately 20 x 14.5 x 16.7 cm. There may be circumferential residual myometrium present, likely a primary endometrial abnormality.

Also noted on CT was an abnormal pelvic mass that appeared uterine in origin, likely uterine leiomyoma. It measured approximately 20 x 14.5 x 16.7 cm (Figure 2).

Biochemical investigations were performed and the diagnosis of pheo was made upon observation of elevated plasma-free metanephrine, urine catecholamines and their metabolites (Table 1).

**Table 1:** Biochemical investigations confirming the pheochromocytoma diagnosis.

	Patient values	Ref Range	Units
Test (24-hour urine chemistry)			
Epinephrine, 24 hr	58	2–24	mcg/24 h
Norepinephrine, 24 hr	2100	15–100	mcg/24 h
Dopamine, 24 hr	517	52–480	mcg/24 h
Urine creatinine	1.27	0.63–2.50	g/24 h
Calc total	2158	26–121	mcg/24 h
24 hr volume	1050		mL
Test (fractionated plasma)			
Total metanephrine	>40,000	<=205	pg/mL
Normetanephrine	>20,000	<=148	pg/mL
Metanephrine	820	<=57	pg/mL
Dopamine	302	<30	pg/mL
Norepinephrine	21,314	217–1109	pg/mL
Epinephrine	286	<95	pg/mL
Total catecholamines	21,600	242–1125	pg/mL

**Figure 3:**
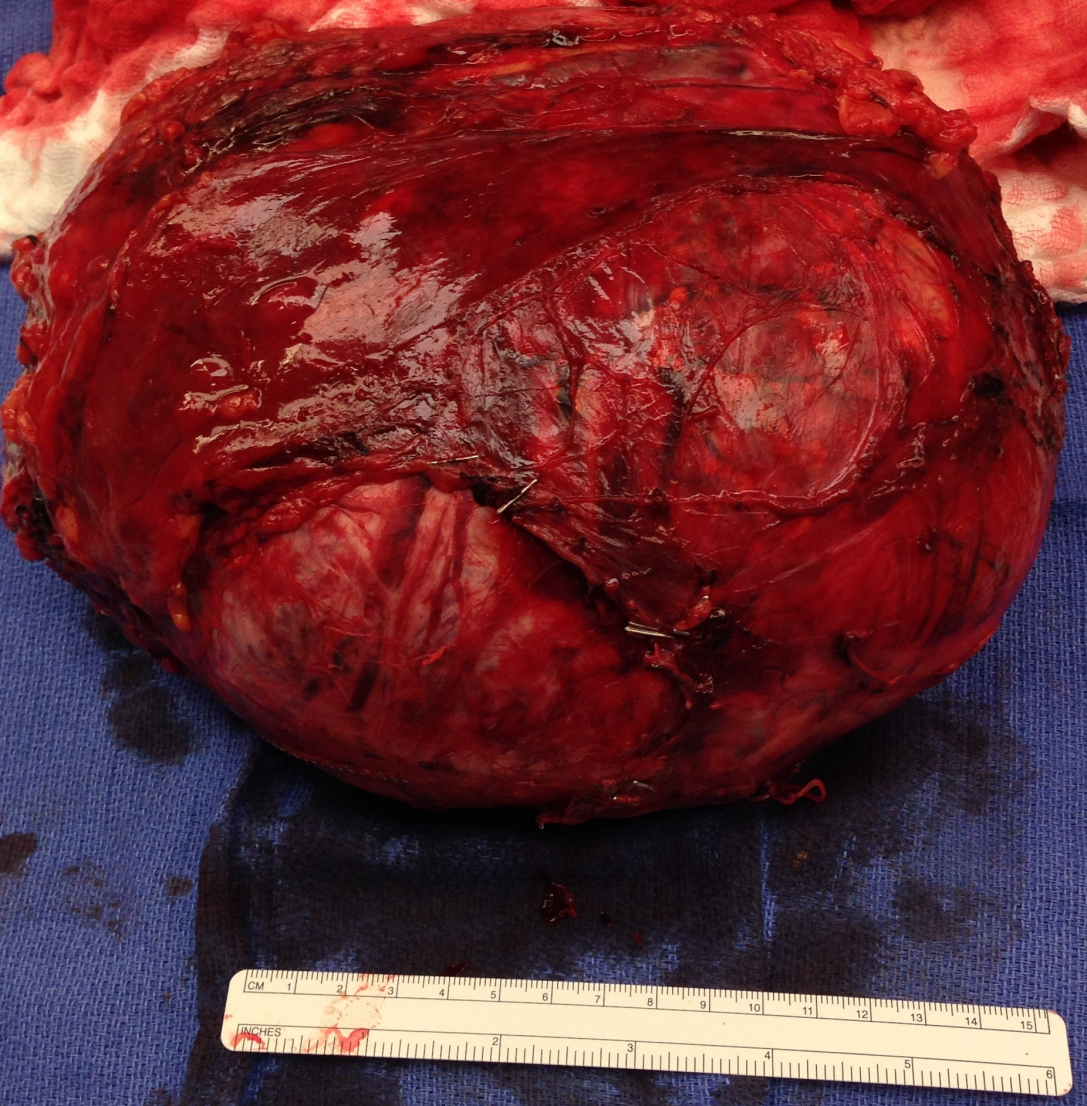
Photograph of the resected right adrenal mass. The surgical specimen revealed a bulging ovoid soft tissue mass measuring 20.5 x 18 x 10 cm and weighing 2,582 g. The soft tissue margin is predominantly smooth with minimal amount of soft tissue adherent to the capsular surface.

Preoperatively, adequate catecholamine blockade was achieved using alpha adrenergic blocker (Terazosin, oral route 3 mg twice a day). An open right adrenalectomy was performed through a right subcostal incision with extension up the midline to the xiphoid. The mass was completely resected en bloc with portion of the right hemidiaphragm (Figure 3). Intraoperatively, there was significant fluctuations in the patient’s blood pressure which was appropriately managed by the anesthesia team (using esmolol, nitroprusside, phenylephrine and norepinephrine drips). Postoperatively, patient progressed well and was discharged home on postoperative day 13.

Pathological examination of the surgical specimen revealed a bulging ovoid soft tissue mass measuring 20.5 x 18 x 10 cm and weighing 2,582 g. Sectioning revealed a soft golden yellow-brown-pink cut surface with prominent hemorrhage and necrosis. Immunohistochemistry revealed tumor cells positive for chromogranin and synaptophysin. Final pathologic diagnosis was pheo with infarct of right adrenal gland with perineural invasion. All inked margins were uninvolved.

Six months after her operation, the patient was scheduled for an elective total abdominal hysterectomy with bilateral salpingo-oophorectomy for symptomatic uterine mass. Pathological examination revealed leiomyoma 17 cm in maximum dimension.

Eleven months from her initial operation, at the time of this report, the patient remained stable and had no symptom to suggest recurrence.

## Discussion

Pheo is an uncommon neoplasm that is derived from chromaffin cells. The annual incidence of pheo is about two million and prevalence in the population is 1:6500 [1]. It occurs in about 0.05% of patients with sustained hypertension, accounting for only about 50% of patients with pheo as only about half of these patients will have paroxysmal hypertension or normotension [1-2]. In about 75% to 85% of adults with pheo, it arises in the adrenal medulla, in 15% to 25%, it arises in the extra adrenal chromaffin tissue, such as the paravertebral ganglia, urinary bladder, posterior mediastinum, and organ of Zuckerkandl [3].

Pheo can present in various ways making it a difficult diagnosis to recognize. Given the fatal complication of untreated pheo, it is very important to screen patients for the disease. The diagnosis of pheo relies on the demonstration of excessive production of catecholamine [4]. The initial screening test is the measurement of either or both fractionated metanephrines in 24-hour urine or plasma free metanephrines. These include the metabolites normetanephrine and metanephrine. The measurement of plasma-free metanephrines has sensitivity exceeding 96% and a specificity between 85% and 100% [5].

After pheo has been diagnosed biochemically, the next step is localizing the tumor using either CT or magnetic resonance imaging (MRI) of the abdomen. The sensitivity of CT scan reaches 85–94% and is approximately 90% for extra-adrenal localizations. The sensitivity of MRI reaches 93–100% [6]. 123I-metaiodobenzylguanidine (MIBG) scintigraphy is a commonly used secondary imaging modality with a high specificity and should be considered in patients with adrenal tumors seen on conventional imaging [5,7]. It can detect additional lesion(s) not detected on conventional imaging in patients with metastatic disease [5]. However, given that MIBG has a low sensitivity, it is not the initial imaging of choice. In a patient with known metastatic disease, 18F-fluorodeoxyglucose (18F-FDG) positron emission tomography (PET)/CT scanning is preferred over MIBG [5,7]. Ideally, functional images technique should be used when there are specific characteristics of the primary tumor that raise suspicion for malignancy.

Genetic testing is selectively performed as part of the diagnostic evaluation for pheo. Pheo is classically associated with three syndromes: von Hippel-Lindau (VHL) syndrome, multiple endocrine neoplasia type 2 (MEN2) and neurofibromatosis type 1 (NF1) [5]. Study shows that patients with pheo diagnosed at an early age (age 30 years) without a family history of or other clinical signs of pheo are more likely to have mutations in VHL or in the succinate dehydrogenase (SDH) subunit genes, specifically SDH B and SDH D mutations compared with tumors in patients with NF1 and MEN2 [5]. In addition, the presence of bilateral adrenal tumors or multifocal extra-adrenal tumors is more likely associated with germline mutations [5]. Mutations in the SDH B and SDH D have been linked to extra-adrenal tumors [7]. In the past 10 years, two additional new pheo associated syndromes have been described: those associated with hypoxia inducible factor two alpha (HIF 2A) and prolyl hydroxylase one or two mutations (PHD 1/2) [8-9].

The management of pheochromocytoma consists of both pharmacological and surgical therapy. The goal of pharmacologic therapy is to control hypertension and associated cardiovascular abnormality in both the preoperative and postoperative settings [4]. Tumor manipulation during surgery can cause serum catecholamine surge resulting in hypertensive crisis and cardiac arrhythmia. Therefore, patients are started preoperatively on phenoxybenzamine, a non-competitive Alpha 1 and Alpha 2 (α1 and α2, respectively) blocker [4]. However, some studies questioned the efficacy of phenoxybenzamine, as some patients pretreated with phenoxybenzamine still had hypertensive crisis during surgery [4,10].

Other pharmacologic alternatives include the use of specific α1 receptor blockers, such as prazosin or terazosin, as this would avoid the reflex tachycardia that is seen with phenoxybenzamine use [4,10]. In addition, given that these agents are short-acting, this would reduce the length of postoperative hypotension [4]. Calcium channel blockers, like cardizem, can also be used as they do not cause severe hypotension and are short-acting [4,10]. These agents can be used safely in patients who are normotensive or have mild hypertension [4]. Beta blockers (β-blockers) have also been used along with alpha blockers to help prevent reflex tachycardia. However, it is important that β-blockers be used after alpha blockade has been optimized because unopposed alpha-adrenergic receptor stimulation can precipitate a hypertensive crisis [4]. Metyrosine, an inhibitor of the enzyme tyrosine hydroxylase, can be used as well to decrease the amount of synthesized catecholamines in the preoperative state and in patients with metastatic and inoperable disease [4].

The ultimate treatment for pheochromocytoma is surgical intervention. According to current guidelines, an open resection is recommended for large adrenal adenomas measuring greater than 6 cm or invasive tumors [7]. Laparoscopic resection can be performed for small, noninvasive tumors [7]. An experienced anesthesiologist and euvolemia are essential. In the immediate postoperative period, watch for hypoglycemia due to increase insulin and possible congestive heart failure due to cardiomyopathy. Following pheochromocytoma excision about 15% will develop recurrence [3]. Long-term follow-up with regular interval office visits, catecholamine checks, and imaging studies are recommended.

## Conclusions

In this report, we presented a case of a 55-year-old super obese female with a giant 20.5 cm pheo with an incidental finding of uterine leiomyoma. Diagnosis of pheo was made by plasma and urine metanephrine levels while imaging was used to localize the mass. Management consisted of providing alpha blocker followed by surgical resection of the mass. Our patient, 11 months after resection, remained stable and disease free. In a patient with multiple surgical indications including pheochromocytoma, as in our case, pheochromocytoma should always be managed first to prevent the risk of adrenergic crisis which can result in increased morbidity and mortality. Multidisciplinary team including cardiologist, endocrinologist, anesthesiologist and the surgeon are needed in the successful management of patients with pheo.
